# Factors associated with the transition from suicidal ideation to suicide attempt in prison

**DOI:** 10.1192/j.eurpsy.2020.101

**Published:** 2020-12-13

**Authors:** Louis Favril, Rory C. O’Connor, Keith Hawton, Freya Vander Laenen

**Affiliations:** 1 Institute for International Research on Criminal Policy, Faculty of Law and Criminology, Ghent University, Ghent, Belgium; 2 Suicidal Behaviour Research Laboratory, Institute of Health & Wellbeing, University of Glasgow, Glasgow, United Kingdom; 3 Centre for Suicide Research, Department of Psychiatry, University of Oxford, Oxford, United Kingdom

**Keywords:** Ideation-to-action, prison, self-harm, suicide, behavioral disinhibition

## Abstract

**Background:**

Although research has identified a wide range of risk factors for suicidal behavior in prisoners, it does not establish who is most likely to act on their suicidal thoughts while incarcerated.

**Methods:**

Self-report data were collected from a random sample of 1,203 adult men incarcerated across 15 prisons in Belgium, who represent 12% of all male prisoners nationwide.

**Results:**

One-third (33%) of participants reported having suicidal thoughts during their incarceration, of whom 26% attempted suicide in prison (9% of all prisoners). Factors independently associated with suicide attempt among prisoners with suicidal ideation were violent offending (adjusted odds ratio [aOR] = 2.64, 95% confidence interval [CI] 1.33–5.23), in-prison drug use (aOR = 2.30, 95% CI 1.25–4.22), exposure to suicidal behavior (aOR = 1.96, 95% CI 1.04–3.68), and a lifetime history of nonsuicidal self-injury (aOR = 1.90, 95% CI 1.08–3.36). While related to suicidal thoughts, markers of psychiatric morbidity and aspects of the prison regime were not associated with the progression to suicide attempt.

**Conclusions:**

Many prisoners who think about suicide do not attempt suicide while incarcerated. Factors associated with suicidal ideation are distinct from those that govern the transition to suicidal behavior. Our findings lend support to the hypothesis that behavioral disinhibition might act as a catalyst in the translation of suicidal thoughts into action.

## Introduction

Suicidal thoughts are common in prisoners [1] and are prospectively associated with suicidal behavior [2]. Meta-analyses have identified suicidal ideation as the strongest of all risk factors for both suicide [3] and self-harm [4] in prisons. Yet, most people who consider suicide will not engage in suicidal behavior [5]. This implies that suicidal ideation is not a sufficient cause nor a sensitive risk marker for suicidal behavior, and that people who do go on to attempt suicide may constitute a discrete class of suicidal individuals. Identifying what factors may precipitate the transition from thought to enactment could elucidate points at which to disrupt this trajectory of risk. In light of this, recent work [6–9] has shown that brain injury, interpersonal violence, nonsuicidal self-injury, childhood adversity, trauma, substance abuse, and certain mental disorders increase the risk of suicide attempt among prisoners with suicidal ideation. Although these risk factors largely mirror those found for behavioral enaction in the general population [10–16], epidemiological data suggest that prisoners are twice as likely as nonincarcerated adults to act on their suicidal thoughts [5–9]. It is possible that factors specific to imprisonment may account for this increased risk, but this hypothesis has not yet been examined. Indeed, a central limitation of the previous four studies [6–9] relates to the assessment of prisoners’ suicidal outcomes on a *lifetime* basis, hence providing limited information concerning what factors may be associated with behavioral enaction *while incarcerated.* Consequently, the potential role of prison-specific influences (e.g., physical and social isolation) has been largely neglected in this emerging line of research—factors known to be associated with prisoners’ risk of suicide more generally [4], but not yet examined within an ideation-to-action framework [17]. Such knowledge would obviously be important for clinicians in determining risk and intervening with suicidal prisoners before they act on their suicidal thoughts. Against this background, we extend our previous work [7] and sought to examine a range of clinical, criminological, and custodial factors associated with in-prison suicide attempt among men who had thought about suicide during their incarceration.

## Methods

### Participants and procedures

A detailed discussion of the sampling procedures and survey methods is outlined elsewhere [18]. Briefly, eligible study participants were all men aged ≥18 years residing in 15 Belgian prisons. Prisons were selected based on their geographical proximity—the Flanders region of Belgium, housing roughly half of all 10,619 prisoners (10,134 [95%] men) nationwide [19]. During the study period (October 2015 to May 2016), a total of 3,636 men were incarcerated in the 15 selected prisons, of whom 1,414 (39%) were randomly selected by computer to participate in the study. Each individual included in this random sample was personally (face-to-face) approached by the first author, a clinical psychologist independent of the prison system. Following written informed consent, participants completed a paper-and-pencil questionnaire in Dutch, French, or English, which was translated by the research team. Ethical approval for the study was granted by the Ethics Committee of Ghent University, Faculty of Law and Criminology. The authors assert that all procedures contributing to this work comply with the ethical standards of the relevant national and institutional committees on human experimentation and with the Helsinki Declaration of 1975, as revised in 2008.

A total of 1,203 men were included in the analyses, equating to an 85% response rate—which tallies with the 78–85% documented in similar large-scale prison studies [6,8,20]. The sample accounts for one-third (33%) of all men physically residing in the 15 selected prisons who were eligible to participate during the data collection period and represents 12% of the average daily population of male prisoners in Belgium at that time [19].

### Measures

#### Demographic and criminological information

Background data were collected on age (years), nationality (Belgian vs. other), and partnership (married/partner vs. other). In the survey, we also asked about relevant criminological variables, including a prior incarceration (no/yes), current custodial status (remand vs. sentenced), time served (categorical), and offense type. The latter variable was recoded into nonviolent (e.g., drug offenses, theft, and fraud) and violent (e.g., murder, manslaughter, and rape) offenses, which is consistent with previous research [6–9]. Two questions assessed prisoners’ employment status in prison (no/yes) and their cell accommodation (shared vs. single cell).

#### Suicidal outcomes

Based on the *British National Psychiatric Morbidity Survey* [1], participants were asked about their lifetime history of suicidal ideation (“Have you ever thought of taking your life, even if you would not actually do it?”) and suicide attempt (“Have you ever made an attempt to take your life?”). Follow-up questions clarified whether this occurred during a (current or previous) period of incarceration. For this paper, we focus on in-prison outcomes during any period of incarceration. Associations with a lifetime history of suicidal ideation and attempt in this sample have been reported elsewhere [7].

#### Self-injury and exposure

A lifetime history of nonsuicidal self-injury was dichotomously assessed by asking participants “Have you ever deliberately harmed yourself in any way, but not with the intention of killing yourself?” [1]. A single item was included to inquire about a family history of suicidal behavior, asking participants whether there was anyone in their family who had ever attempted or died by suicide (no/yes). Another question assessed whether prisoners were confronted with or witnessed a suicide or suicide attempt by a fellow prisoner during their incarceration (no/yes).

#### Psychiatric morbidity and drug use

A self-reported diagnosis of a mental disorder was assessed by asking participants “Have you ever been told by a mental health professional, such as a psychiatrist or psychologist, that you had one or more of the following mental disorders?” followed by a comprehensive list of diagnostic labels (including mood, anxiety, psychotic, eating, personality, and substance use disorders). The wording of the question and choice of a self-report measure of lifetime psychiatric diagnoses are consistent with previous research in prisoners [9,20]. Following the *New South Wales Inmate Health Survey* [21], participants were also asked about in-prison use of illicit drugs (excluding alcohol) and currently prescribed psychotropic medication.

#### Quality of prison life

Prisoners’ perceptions of their quality of life in prison were collected using the *Measuring the Quality of Prison Life* survey, a validated self-report instrument asking prisoners directly about the prison regime and relationships within prison [22]. We assessed five prison dimensions through 23 statements which participants (dis)agreed with on a five-point Likert scale ranging from 1 (strongly disagree) to 5 (strongly agree): *personal autonomy* (four items; e.g., “I have no control over my day-to-day life in here”), *physical safety* (five items; e.g., “I feel safe from being injured, bullied, or threatened by other prisoners in here”), *decency* (four items; e.g., “Prisoners spend too long locked up in their cells in this prison”), *outside contact* (three items; e.g., “I am able to receive visits often enough in this prison”), and *staff relationships* (seven items; e.g., “Overall, I am treated fairly by staff in this prison”). Responses were recoded so that all items were scored in a positive direction, with lower scores indicating a more negative judgment of the particular prison dimension. Cronbach’s *α* ranged from 0.49 (personal autonomy) to 0.84 (staff relationships).

#### Social support

Prisoners’ self-perceived social support was assessed using the *Social Support Scale*, a seven-item instrument previously used in prison research [1,23]. Each item (e.g., “There are people I know who can be relied on, no matter what happens”) has three response options scored between 1 (not true) and 3 (certainly true). Overall scores ranged from 7 to 21, with higher scores suggesting higher levels of perceived social support (Cronbach’s *α* = 0.91). Composite scores of 17 or less were used as an indicator of poor social support [23].

#### Statistical analysis

The analytical plan consisted of two consecutive phases. First, we examined which clinical, criminological, and custodial factors were associated with *suicidal ideation* while incarcerated among the total sample of prisoners (*n* = 1,203). Second, we examined which of these factors were associated with in-prison *suicide attempt* among participants reporting suicidal ideation (*n* = 399). For both phases, bivariate (*χ* ^2^ tests for categorical variables and independent-samples *t* tests for continuous variables) and multivariate (binary logistic regression) analyses were conducted. The latter analysis controlled for demographic variables (age, nationality, and partnership) and all other factors (regardless of whether they were significant at the bivariate level) in order to identify independent contributions. Crude (OR) and adjusted odds ratios (aORs) are presented as estimates of the strength of bivariate and multivariate associations, respectively. A missing values analysis was conducted, showing that variables contained few missing cases, with less than 5% missing values for all individual items. This was deemed ignorable missingness, and listwise deletion was used to handle missing cases for all analyses [7,9]. All analyses were done in SPSS version 26, and statistical significance was set at *p* < 0.05.

## Results

### Sample characteristics

The mean age of participants was 37.7 years (standard deviation [SD] = 11.9, range 18–77) and 72.1% were of Belgian nationality. One-third (34.3%) was currently on remand (awaiting trial), with the other 790 men (65.7%) being sentenced. A quarter (25.5%) of prisoners were charged with, or convicted of, a violent offense. More than half (58.9%) had a prior history of incarceration. At the time of assessment, approximately one-third of participants had been in prison for less than 6 months (38%), 6 months up to 3 years (32%), and more than 3 years (30%). Further details on participants’ clinical characteristics and custodial factors are listed in Table 1.

**Table 1.** Sample characteristics and associations with suicidal outcomes in prison.

	Total sample	Suicidal ideation (*n* = 1,203)		Suicide attempt (*n* = 399)	
	*n* (%) or M (SD)	OR (95% CI)	aOR (95% CI)	OR (95% CI)	aOR (95% CI)
*Criminological*					
Prior incarceration	709 (58.9)	0.98 (0.77–1.25)	0.70 (0.50–0.98)	1.00 (0.64–1.58)	0.76 (0.41–1.40)
Sentenced status	790 (65.7)	1.38 (1.07–1.79)	0.90 (0.60–1.37)	2.26 (1.30–3.93)	1.97 (0.86–4.53)
Duration incarceration					
<6 months	457 (38.0)	0.57 (0.43–0.76)	1.06 (0.64–1.75)	0.40 (0.23–0.69)	0.70 (0.28–1.74)
6–36 months	385 (32.0)	0.59 (0.44–0.80)	0.96 (0.65–1.42)	0.53 (0.30–0.91)	0.62 (0.31–1.23)
>36 months	361 (30.0)	1.00 (reference)	1.00 (reference)	1.00 (reference)	1.00 (reference)
Violent offense	294 (25.5)	2.05 (1.56–2.69)	1.64 (1.13–2.40)	1.71 (1.08–2.72)	2.64 (1.33–5.23)
*Clinical*					
Psychotropic medication	414 (34.4)	2.91 (2.26–3.74)	1.59 (1.13–2.23)	2.24 (1.41–3.56)	1.17 (0.63–2.16)
Psychiatric diagnosis	539 (44.8)	3.04 (2.37–3.90)	1.86 (1.32–2.64)	2.12 (1.28–3.49)	1.46 (0.75–2.83)
Drug use in prison	422 (35.1)	2.21 (1.72–2.83)	1.19 (0.83–1.71)	2.72 (1.71–4.33)	2.30 (1.25–4.22)
Nonsuicidal self-injury	199 (16.5)	3.85 (2.81–5.27)	2.36 (1.59–3.50)	2.70 (1.69–4.32)	1.90 (1.08–3.36)
Family history	297 (24.7)	2.36 (1.80–3.09)	1.41 (1.00–1.98)	1.17 (0.73–1.85)	0.85 (0.48–1.52)
*Custodial*					
Suicide exposure	575 (47.8)	2.64 (2.06–3.38)	2.40 (1.73–3.33)	1.90 (1.16–3.12)	1.96 (1.04–3.68)
Single cell occupancy	596 (50.3)	1.36 (1.07–1.74)	1.25 (0.89–1.76)	1.26 (0.80–1.99)	1.16 (0.62–2.15)
Prison employment	644 (53.9)	0.66 (0.52–0.84)	0.62 (0.45–0.85)	0.69 (0.44–1.09)	0.68 (0.38–1.22)
Poor social support	558 (47.4)	1.47 (1.15–1.88)	1.68 (1.02–2.78)	1.31 (0.83–2.06)	1.43 (0.81–2.54)
Quality of prison life					
Personal autonomy	2.78 (0.76)	0.56 (0.47–0.66)	0.75 (0.59–0.95)	0.99 (0.74–1.32)	1.29 (0.84–1.97)
Outside contact	3.00 (0.98)	0.74 (0.65–0.84)	0.80 (0.67–0.95)	0.85 (0.67–1.07)	0.89 (0.64–1.23)
Staff relationships	2.84 (0.88)	0.74 (0.65–0.86)	1.07 (0.85–1.36)	0.98 (0.76–1.28)	0.78 (0.49–1.27)
Physical safety	3.20 (0.82)	0.49 (0.42–0.58)	0.61 (0.49–0.75)	0.78 (0.60–1.03)	0.96 (0.66–1.41)
Decency	2.64 (0.76)	0.73 (0.62–0.86)	1.06 (0.80–1.39)	0.78 (0.58–1.05)	0.89 (0.53–1.48)

Abbreviations: aOR, adjusted odds ratio (adjusted for age, nationality, partnership, and all other variables in the multivariate analysis); CI, confidence interval; SD, standard deviation.

### Prevalence estimates

The prevalence of suicidal ideation and suicide attempt while in prison was 33.2% (95% confidence interval [CI] 30.5–35.8) and 9.1% (95% CI 7.4–10.7), respectively. Five participants who attempted suicide did not report suicidal ideation. A quarter (26.1%, 95% CI 21.7–30.4) of the 399 participants with suicidal ideation had made a suicide attempt while incarcerated.

### Associations with suicidal ideation

In the total sample (*n* = 1,203), all but one (prior incarceration) variables were bivariately associated with suicidal ideation in prison (Table 1). Of those significant, odds ratios ranged from 1.36 (single cell accommodation) to 3.85 (nonsuicidal self-injury) for positive associations and from 0.74 (both outside contact and staff relationships) to 0.49 (physical safety) for negative associations.

In the multivariate analysis (Table 1), violent offending increased the odds of suicidal ideation by 64% (aOR = 1.64, 95% CI 1.13–2.40), whereas a prior incarceration was negatively associated with this outcome (aOR = 0.70, 95% CI 0.50–0.98). Of the clinical variables, psychotropic medication (aOR = 1.59, 95% CI 1.13–2.23), psychiatric diagnosis (aOR = 1.86, 95% CI 1.32–2.64), and nonsuicidal self-injury (aOR = 2.36, 95% CI 1.59–3.50) were all independently associated with suicidal ideation in prison, but drug use was not (aOR = 1.19, 95% CI 0.83–1.71). Most of the custodial variables were significantly related to experiencing suicidal thoughts while incarcerated, including physical safety (aOR = 0.61, 95% CI 0.49–0.75), working activity (aOR = 0.62, 95% CI 0.45–0.85), personal autonomy (aOR = 0.75, 95% CI 0.59–0.95), outside contact (aOR = 0.80, 95% CI 0.67–0.95), and poor social support (aOR = 1.68, 95% CI 1.02–2.78). This was also the case for exposure to suicidal behavior in prison (aOR = 2.40, 95% CI 1.73–3.33).

### Associations with suicide attempt

When considering the subsample of prisoners with suicidal ideation (*n* = 399), bivariate analyses show that several criminological (violent offending and being sentenced) and clinical (psychotropic medication, psychiatric diagnosis, drug use, and nonsuicidal self-injury) variables increased the odds of suicide attempt in prison (OR range 1.71–2.72; Table 1). Duration of incarceration was negatively associated with this outcome. None of the prison-specific variables was bivariately associated with the transition from suicidal ideation to suicide attempt, except for exposure to suicidal behavior while incarcerated (OR = 1.90, 95% CI 1.16–3.12).

Only four factors were independently associated with suicide attempt among prisoners with suicidal ideation in the multivariate analysis with effect sizes clustered around 2 (Table 1). Violent offending (aOR = 2.64, 95% CI 1.33–5.23), in-prison drug use (aOR = 2.30, 95% CI 1.25–4.22), exposure to suicidal behavior of other prisoners (aOR = 1.96, 95% CI 1.04–3.68), and a lifetime history of nonsuicidal self-injury (aOR = 1.90, 95% CI 1.08–3.36), each contributed uniquely (relative to the other variables in the model) to the transition from suicidal ideation to suicide attempt in prison.

## Discussion

To our knowledge, this study is the first to investigate factors associated with the transition from suicidal ideation to suicide attempt while incarcerated. We did so among a random sample of 1,203 men, of whom 9% attempted suicide in prison—a figure in the range of previous estimates (9–13%) found among male prisoners in Europe [24–27]. Our analyses highlight two key findings. First, factors characterized by behavioral disinhibition—drug use, violent offending, and nonsuicidal self-injury—each doubled the odds of suicide attempt among prisoners with suicidal ideation, as did exposure to suicidal behavior. Second, while related to suicidal thoughts, markers of psychiatric morbidity and aspects of the prison regime were not associated with the transition to suicide attempt. Both of these findings warrant additional comment.

Our results are in keeping with community-based findings that behaviors which might be labeled as *dysregulated* (including substance use, interpersonal violence, and self-injury) [28] increase the risk of suicide attempt in the context of suicidal ideation [11–15]. Deficits in executive functioning could be a shared diathesis underlying these associations, in that individuals with reduced inhibition might have difficulty resisting the urge to act on suicidal thoughts [29,30]. Indeed, research suggests that prisoners who are violent (toward others or oneself) and use drugs have lower impulse control than their incarcerated counterparts [31–33], which might explain their heightened propensity to behavioral enaction evidenced by this study. Reinforcing this assumption, recent population-based studies [34,35] suggest that impulsivity is one of the distinguishing factors between youth who consider and attempt suicide. Together, our findings lend support to the hypothesis that behavioral disinhibition might act as a catalyst in the transition from thought to enactment [16].

Markers of psychiatric morbidity, both current (medication) and historical (diagnosis), were not independently associated with behavioral enaction in prison, although they increased the odds of prisoners experiencing suicidal thoughts while incarcerated. Our data align with epidemiological [36–39] and meta-analytical [10] evidence indicating that virtually all mental disorders increase the risk of subsequent suicidal ideation, but only a select few―those characterized by anxiety and poor impulse control―predict the transition to suicide attempt. This finding was recently replicated in a representative national sample of 1,212 New Zealand prisoners [8], in that most mental disorders were not associated with suicide attempt above and beyond their relationship with suicidal ideation. Although we did not examine individual disorders in the current study, which limits a more granular approach to determine their unique contributions, our results suggest that psychiatric morbidity in the broad sense might affect the cognitive (ideation) rather than the behavioral (attempt) spectrum of suicide risk in prisoners.

Similarly, while related to suicidal thoughts, aspects of the prison regime were not associated with the transition to suicide attempt. Previous studies have documented that prisoners’ negative perceptions of the correctional climate (relating to autonomy, safety, meaningful activities, and relationships) are associated with poor mental health [40–42]. Although a recent meta-analysis [4] and several qualitative investigations [43,44] support the fact that institutional factors and prison experiences influence one’s risk of suicide, no studies to date have examined their differential associations with distinct stages of the suicidal process. Pending replication, our data suggest that factors relating to the prison environment, and the subjective experience thereof, contribute to the development of suicidal thoughts but do not impact on the progression to suicide attempt.

Consistent with studies highlighting that exposure to suicidal behavior of others is associated with the transition from ideation to attempt in youth [11,35], we found that prisoners exposed to suicidal behavior of incarcerated peers were twice as likely to act on their suicidal thoughts relative to those with no such exposure. This is an important finding given the evidence of spatiotemporal clustering of suicide [45] and self-harm [46] in prisons. Potential mechanisms underlying this relationship could include imitation and social learning [47]. For example, exposure to suicidal behavior of others could provide a behavioral model for susceptible prisoners, increasing the likelihood that thoughts of suicide are acted on. Exposure might also increase the salience and acceptability of suicidal behavior through increased awareness of suicide as an option or knowledge of methods [11]. Alternatively, assortative relating [47] might be imposed in the sense that prisoners with similar vulnerability profiles (e.g., violent offenders and drug users) are likely to be housed in the same prison type or wing. Although more research is needed to better understand the psychological processes at play, this study strengthens the evidence of exposure as a risk factor for suicidal behavior [48].

In summary, our data suggest that factors associated with suicidal ideation are distinct from those that govern the progression from thought to enactment in prison, which aligns with recent ideation-to-action theories of suicide [17]. Specifically, markers of psychiatric morbidity and aspects of the prison regime may best be conceptualized as risk factors for suicidal ideation rather than for suicide attempt. Instead, exposure and behaviors characterized by disinhibition were independently associated with the transition from suicidal ideation to suicide attempt while incarcerated. These factors all reflect painful and provocative experiences, which have been hypothesized to increase one’s capability to engage in suicidal behavior [49].

### Limitations

Our findings should be interpreted in light of several methodological constraints. The main limitation of this study concerns its cross-sectional design, which inherently precludes any causal inferences. Though cross-sectional data can yield insights into relationships between variables, they cannot answer whether the risks associated with suicide attempt are causal or coincidental, or whether the outcome might *itself* be a cause of other behaviors (e.g., drug use). Moreover, several variables in our study covered different time periods (i.e., current, while incarcerated, and lifetime), and we examined outcomes during any period of incarceration, which further limits assertions about the temporality of observed associations. As a result, it will be crucial to evaluate the consistency of our results in future prospective studies that carefully document the time of onset of each predictor and outcome. Furthermore, our analyses relied upon retrospective self-report data, which are vulnerable to biased recall and social desirability [5]. Next, we adopted a dichotomous assessment for suicidal outcomes, which may bias results toward an inflation of prevalence estimates due to misclassification [50]. Future studies should assess characteristics of suicidal thinking (e.g., recency, frequency, uncontrollability, severity, and planning) as these have been shown to predict the transition to suicide attempt [36,51]. In a similar vein, most clinical variables in our analyses were assessed by single-item questions, as opposed to more fine-grained and psychometrically validated scales. Specifically, self-report approaches to clinical diagnosis are not without problems, and clinician-administered diagnostic interviews would be a more accurate approach [52]. Finally, since differences exist between risk factors for fatal and nonfatal suicidal behaviors [53], the current findings may not be generalizable to prisoners who die by suicide [54].

### Implications

Suicidal thoughts and attempts are highly prevalent in prisoners worldwide [6–9], which are among the strongest predictors of death by suicide [3]. Knowledge of what factors may be implicated in the trajectory toward suicide can improve prevention efforts in this high-risk population.

Although extant research [3,4] has identified a wide range of risk factors for suicidal behavior in prisoners, it does not establish who is most likely to act on their suicidal thoughts. This distinction is important since only a proportion of individuals who consider suicide will ultimately engage in suicidal behavior [5]. We found that three-quarters of prisoners who experienced suicidal thoughts during their incarceration did not attempt suicide, which implies that progression along the suicidal continuum can be halted. Therefore, timely strategies aimed at reducing the likelihood of prisoners acting on their suicidal thoughts should be implemented. A critical first step includes the identification of those at risk for suicide—a challenge faced by many clinicians working with prisoners. The current findings provide some guidance on the types of questions that clinicians might want to address when a prisoner discloses having thoughts of suicide. Screening for substance use, self-injury, and violent behavior could be beneficial, as could a closer inspection of one’s propensity to be impulsive. Our data further show that dysregulated behaviors may present actionable targets for psychosocial intervention. Dialectical and cognitive behavior therapy could be promising in this respect [16], but current evidence of effectiveness in custodial settings is weak [55], and safety planning should be considered. Furthermore, given the association between exposure and suicide attempt, interventions following suicidal behavior in prison should extend beyond the individual prisoner to others in the same wing or prison who could be at risk.

We found that variables relating to prisoners’ mental health and their perceptions of the prison climate were not uniquely associated with suicide attempt. This finding, however, does not suggest that these factors are unimportant in the etiology or prediction of suicidal behavior [4], but merely that they are not especially useful in determining who is likely to act on their suicidal thoughts in prison. From a preventive point of view, interventions should equally be targeted at addressing the development of suicidal ideation [18]. Accordingly, mental health care remains a central component of any suicide prevention strategy in prison [56], which should be complemented by environmental interventions and changes to the prison regime—including measures aiming to promote autonomy, purposeful activity, and social support [57]. The latter approach is further supported by neuropsychological evidence suggesting that the impoverished custodial environment may exert a negative effect on prisoners’ self-control [58].

In closing, further research is needed to better understand the mechanisms through which prisoners come to think about suicide and subsequently decide (not) to act on suicidal thoughts. Prospective studies should build on our findings and explore whether the identified factors indeed *predict* suicidal outcomes during the course of imprisonment, with particular focus on impulse control as a possible intervention target. A more in-depth appreciation of the process of behavioral enaction has the potential to improve detection, management, and prevention of suicide risk in prisoners.

## Data Availability

The data that support the findings of this study are not publicly available.
